# Effect of vitamin energy drinks on relieving exercise-induced fatigue in muscle group by ultrasonic bioimaging data analysis

**DOI:** 10.1371/journal.pone.0285015

**Published:** 2023-06-26

**Authors:** Xindi Wang, Mengtao Xu

**Affiliations:** 1 School of Aerospace, Harbin Institute of Technology, Harbin, Heilongjiang, China; 2 China Basketball College, Beijing Sport University, Beijing, Beijing, China; Wuhan University of Technology, CHINA

## Abstract

**Objective:**

This work was aimed to analyze the effect of vitamin energy drink on muscle fatigue by surface electromyography (SEMG) and ultrasonic bioimaging (USBI).

**Methods:**

20 healthy men were selected to do increasing load fatigue test. Surface electromyographic signals and ultrasonic biological images were collected based on wavelet threshold function with improved thresholds. Time domain and frequency domain characteristic integrated electromyography (IEMG), root mean square amplitude (RMS), average power frequency (MPF), and surface and deep muscle morphological changes were analyzed. Hemoglobin concentration (HB), red blood cell number (RBC), mean volume of red blood cell (MCV), blood lactic acid (BLA), malondialdehyde (MDA), and phosphocreatine kinase (CK) were measured.

**Results:**

1) the Accuracy (94.10%), Sensitivity (94.43%), Specificity (93.75%), and Precision (94.07%) of the long and short-term memory (LSTM) specificity for muscle fatigue recognition were higher than those of other models. 2) Compared with the control group, the levels of BLA, MDA, and CK in the experimental group were decreased and HB levels were increased after exercise (*P* < 0.05). 3) IEMG and RMS of the experimental group were higher than those of the control group, and increased with time (*P* < 0.05). 4) The mean amplitude of the response signal decreased with time. Compared with the control group, the surface muscle thickness, deep muscle thickness, total muscle thickness, contrast, and homogeneity (HOM) decreased in the experimental group; while the angular second moment (ASM) and contrast increased, showing great differences (*P* < 0.05).

**Conclusion:**

Surface electromyographic signal and ultrasonic biological image can be used as auxiliary monitoring techniques for muscle fatigue during exercise. Drinking vitamin energy drinks before exercise can relieve physical fatigue to a certain extent and promote the maintenance of muscle microstructure.

## 1. Introduction

Muscle fatigue is a very common physiological phenomenon, and it is also the process of reducing the maximum muscle output power. The fatigue of muscle tissue is closely related to the intensity of muscle exercise. If the degree of muscle fatigue exceeds the maximum load level, the fibrous filaments in the muscle will separate or break, eventually leading to muscle injury [1]. Severe or persistent fatigue will result in reduced immune levels and endocrine system disorders, as well as organic diseases when serious, affecting human health. Exercise-induced fatigue (EIF) is often accompanied by changes in metabolites, cell metabolism regulation enzymes, hormones, antioxidant enzymes, and other substances, thus leading to the imbalance of environmental homeostasis in vivo [2]. The intake of energy drinks can replenish the water, energy, and electrolytes lost by the body, improve the exercise ability, and effectively relieve the fatigue after exercise [3]. Vitamins are essential for growth, metabolism, and development; appropriate vitamin supplementation can remarkably improve fatigue [4]. EIF is the reduction of the working ability of body caused by exercise, and is also a normal physiological phenomenon can be restored after rest and adjustment [5]. Fatigue objectively refers to the general or local discomfort due to body activities or muscle activities, while subjective fatigue is the fatigue state caused by psychological activities and other factors.

Currently, the methods used to evaluate muscle fatigue severity consist of the maximum impedance of muscle tissue and other physiological manifestations [6]. Biochemical indicators commonly used for fatigue severity evaluation include blood lactic acid (BLA) and sarcoplasmic bivalent calcium ion activity [7]. Physical indicators are composed of heart rate, muscle strength, muscle thickness, surface electromyography (SEMG) signal, and so on [8]. SEMG signal is the electrical change that occurs along with the action potential conduction of muscle fibers when skeletal muscle is excited, so it can be used to evaluate the fatigue severity of muscle tissue. Electromyography and muscle ultrasonic bioimaging (USBI) signals have also been widely applied in the evaluation of skeletal muscle function [9]. When USBI images are taken to detect muscle structure changes, the image signals will not be disturbed by artifacts of electrical stimulation. However, it is very important to extract useful information from SEMG signals and USBI images to evaluate the severity of muscle fatigue. At present, there are relatively few studies on the evaluation of vitamin energy drinks (VEDs) to improve muscle fatigue.

**Fig 1:**
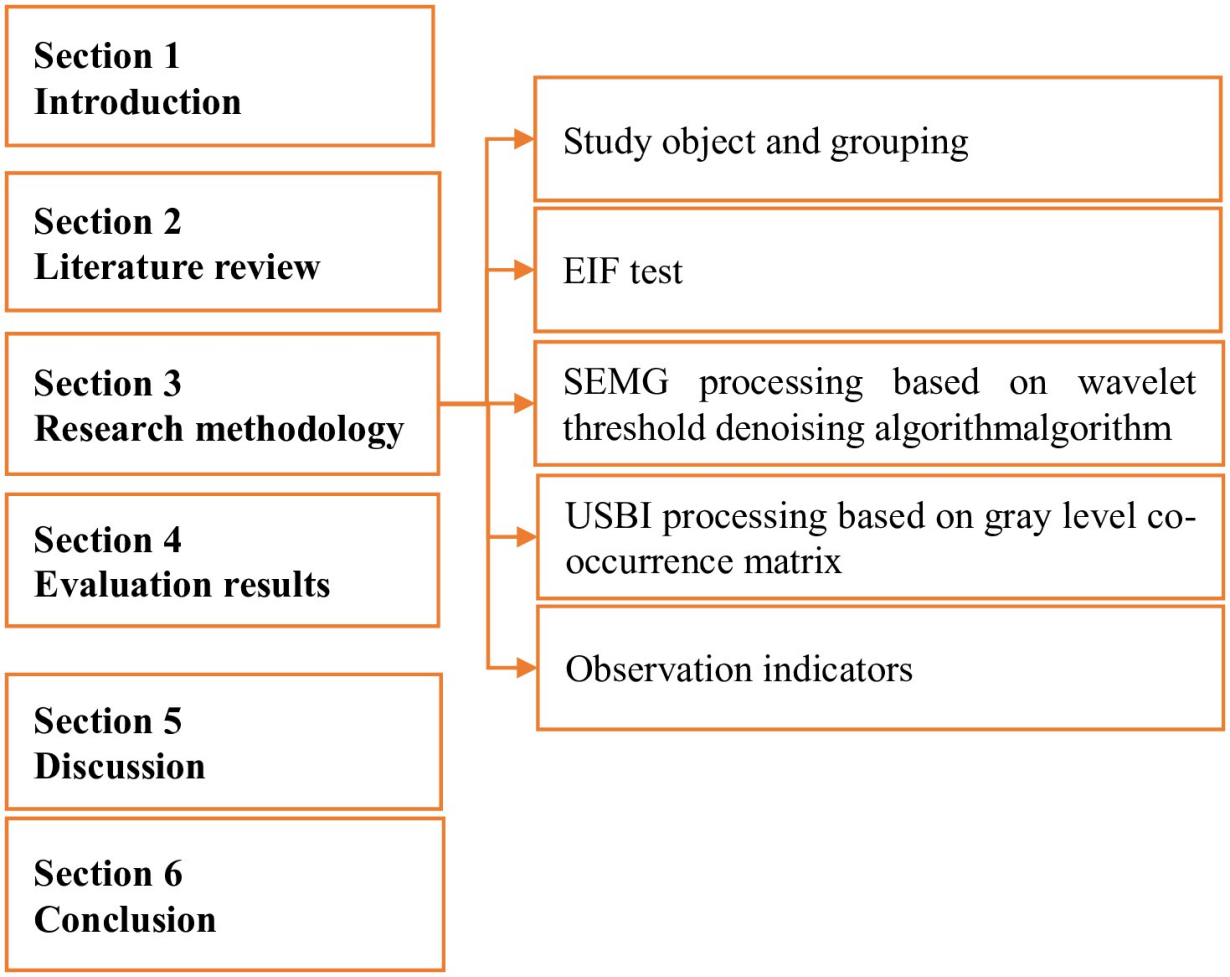
Introduction to the paper process.

This was aimed to understand the effect of VEDs on EIF of muscle groups. In this study, healthy subjects were enrolled for incremental load fatigue test, and changes in SEMG signals and muscle USBI images were collected. The process of the paper is shown in Fig 1. The first section is introduction, which introduces the research background and points out the purpose and significance. Section 2 “Literature review” summarizes the research on this study. Section 3 points out the source of patients participating in this study and the grouping method, describes the research methods used in this study, surface electromyographic signal and muscle ultrasonic biological image processing, processing effect evaluation methods. The fourth section evaluates the results and introduces the research results obtained from the above experimental methods. The fifth section discusses and analyzes the results obtained from this study based on the relevant studies of other scholars. Section 6 summarizes the results and limitations of the study, and points out the methods of future research and the significance of this study.

## 2. Literature review

Muscle fatigue is a common physiological manifestation of athletes in sports training. Vitamin deficiency will lead to metabolic disorders, physiological function, water-soluble vitamins, and exercise fatigue is closely related. Tardy et al. (2020) [10] concluded that B vitamins, vitamin C, iron, magnesium, zinc, and other micronutrients can participate in fatigue performance through biochemical basis and action at the cellular level. Hureau et al. (2021) [11] studied the effects of intravenous vitamin C injection on neuromuscular fatigue and exercise tolerance of patients with chronic obstructive pulmonary disease, and found that vitamin C can improve patients’ REDOX balance and exercise dyspnea, and reduce fatigue development by increasing superoxide dismutase activity. Surface electromyography and ultrasonic bioimaging can be used to evaluate muscle fatigue status. Xu et al. (2022) [12] used surface electromyographic signals to monitor the fatigue state of upper limb movement, developed a surface electromyographic recognition model, and found that the effectiveness of fatigue threshold algorithm combined with biomechanics in fatigue state recognition was significantly improved. Makaram et al. (2020) [13] collected the changes of surface electromyographic signals during dynamic curling movement. The frequency and amplitude of surface electromyographic signals were different for subjects with different exercise fatigue degrees, which can be used in the auxiliary diagnosis of neuromuscular diseases. It can be known that the application of ultrasonic biological image to the evaluation of muscle fatigue is breakthrough research, which can directly reflect the fatigue state of muscle tissue. However, in the process of conventional surface electromyographic signal and ultrasonic biological image acquisition, there is a lot of noise interference, which affects the extraction of useful information and disease diagnosis.

With the wide application of artificial intelligence in various fields, including long short-term memory (LSTM) neural network and support vector machine (SVM) [14–16]. Ashraf et al. (2023) [17] adopted a new denoising algorithm based on variational mode decomposition, and used SOFT iterative interval threshold to process surface and intramuscular electromyography. It was found that the combination of the IIT threshold technique and the SOFT threshold operator based on VMD produced the best denoising results while retaining the original signal characteristics. Yang et al. (2021) [18] proposed an improved flexible analysis wavelet transform (FAWT) algorithm for denoising SEMG signals, proving that the improved FAWT algorithm has a good denoising effect on SEMG. Wang et al. (2022) [19] proposed an accurate feature extraction method for single-channel SEMG signals to improve the accuracy of SEMG signals in different muscles. All the above studies show that artificial intelligence algorithm has a good application prospect in surface electromyographic signal and ultrasonic biological image data processing. Therefore, based on the artificial intelligence technology, surface electromyographic signal and ultrasonic biological image data were used to carry out relatively few studies on the effect of vitamin functional drinks on muscle exercise fatigue state in this work.

## 3. Research methodology

### 3.1 Research objects and their grouping

20 healthy male subjects were selected, aged 20–28 years old, 170–185 cm tall, 60–80 kg weight, body mass index 21.0–26.0 kg/m2. Inclusion criteria: they had (1) a great physical health; (2) no cardiovascular disease; and (3) no serious muscle injury. Exclusion criteria were composed of the followings: (1) Those had an intake of caffeine, nicotine, or alcohol within 24 hours before the experiment. (2) Those had participated in strenuous exercise before the experiment. (3) Those were allergic to drinks or food used in the study. (4) Those suffered from heart diseases, epilepsy, and other diseases that were easy to induce an onset after intense exercise. Subjects had fully understood the objective, processes, and potential risks of this study, and voluntarily participated in this project. The subjects were randomly divided into control (Ctrl) group and experimental (Expt) group, with 10 people per group. The mean age of those in Ctrl group was 22.3±2.09 years old, the mean height was 173.1±4.8 cm, the mean weight was 65.4±7.2 kg, and the mean body mass index was 23.7± 2.1 kg/m2. The mean age of Expt group was 23.0±2.12 years old, besides, the mean height, mean height, and mean body mass index was 174.2±3.5 cm, 65.9±6.8 kg, and 23.3±2.4 kg/m2, respectively. There was not any significant difference in general data between groups (P>0.05). The experiment was carried out in accordance with the technical specifications for the inspection and evaluation of health food issued by Ministry of Health. The subjects in Ctrl group drank 250 mL purified water before the experiment, while those in Expt group drank VED before the experiment. The effects of this beverage to relieve EIF was analyzed and discussed from the aspects of blood biochemical indicators and human body function.

### 3.2 EIF test

The EIF test of subjects was conducted using the incremental load fatigue experiment. The subjects were warmed up 5 minutes before taking the Monark constant-power bicycle. After the start of the exercise, the initial constant power was set as 75 W, and the test time of each stage was 1 minute. The exercise was carried out with the pedaling power at a rate of 65 r/min. The experiment was terminated when the subject’s heart rate reached 85% of the maximum safe heart rate, or when the muscles were struggling to maintain the pedaling power at 65 r/min.

The SEMG signals and muscle USBI images were processed on a computer with 8 Gb memory under Intel Core TM i7-9800 system. Matlab R2020a software was applied to denoise SEMG signals, extract features, and build the muscle fatigue recognition model, then the texture features of muscle USBI images were extracted.

### 3.3 SEMG processing based on wavelet threshold denoising algorithm

There were a lot of noise signals in the process of SEMG signal acquisition. Noise elimination was very important for extracting real signals for subsequent analysis. Wavelet threshold denoising algorithm had the characteristic of excellent denoising effect, so it was applied in removal of signal noises [20]. In the wavelet threshold denoising algorithm, the common functions were soft threshold, hard threshold, adaptive threshold, etc. [21]. In this work, the wavelet threshold denoising algorithm with improved threshold function, which was between soft threshold and hard threshold, was selected for SEMG signal denoising as the Eq (1).


$$W_{j.k}=\left\{\begin{array}{c} w_{j,k}-sign\left(w_{j,k}\right)\frac{k\lambda^{k+1}}{\left(k+1\right){\left|w_{j,k}\right|}^{k}}+sign\left(w_{j,k}\right)\sqrt{w_{j,k}{}^{2}-\lambda^{2}},\;\;\left|w_{j,k}\right|\geq \lambda \\ \left[\left(\frac{1}{k+1}\right)sign\left(w_{j,k}\right){\left|w_{j,k}\right|}^{k+1}\right]/\lambda^{k},\;\;\left|w_{j,k}\right|<\lambda \end{array}\right.$$ (1)

In the above equation, *w*_*j*,*k*_ represented the wavelet coefficient; *λ* stood for threshold, $\lambda =\frac{\left(\left|w_{j,k}\right|\right)median}{0.6745}\sqrt{2∙log(N)},\;\left(\left|w_{j,k}\right|\right)median$ was the median value of wavelet decomposition coefficient.

The time domain features of SEMG signal processed by denoising were IEMG and RMS amplitude, which were worked out as Eqs (2) and (3).


$$IEMG=\int_{t}^{t+T}\left|x\left(t\right)\right|dt$$ (2)

$$RMS=\sqrt{\left(\frac{1}{T}\int_{t}^{t+T}x^{2}\left(t\right)dt\right)}$$ (3)

Frequency features of SEMG signals were calculated by short-time Fourier transform as Eq (4).


$$MPF=\frac{\int_{min}^{max}f\cdot PS\left(f\right)df}{\int_{min}^{max}PS\left(f\right)df}$$ (4)

In the above equations, *PS* was the power spectrum of SEMG signal; *min* was the minimum frequency of signal bandwidth in frequency domain; *max* was the maximum frequency of signal bandwidth in the frequency domain.

Long short-term memory (LSTM) [22, 23] was utilized to identify muscle fatigue state. The number of layers of the LSTM model is set as input layer, hidden layer, and output layer through the query of relevant research literature, and the unit is 100. The loss function was real-time recurrent learning, and the optimizer was Adam function. The activation function was ReLU, the batch size was 70, and the initial learning rate was 0.001. To raise the recognition performance of the model, the early stopping mechanism was introduced to set the stopping training time of the network model, so as to give the generalization ability of the model.

### 3.4 USBI processing based on gray co-occurrence matrix

The positions of surface muscle and deep muscle in muscle USBI image were manually selected, and muscle contractions were marked and tracked. Normalization of pre-contraction and post-contraction dimensions of muscles was performed as Eq (5).


$$R\left(d\right)=\frac{\sum_{m=0}^{M-1}\sum_{n=0}^{N-1}\left\{\left[x\left(m,n\right)-\bar{x}\right]\left[y\left(m,n\right)-\bar{y}\right]\right\}}{\sqrt{\sum_{m=0}^{M-1}\sum_{n=0}^{N-1}{\left\{\left[x\left(m,n\right)-\bar{x}\right]\right\}}^{2}}\sqrt{\sum_{m=0}^{M-1}\sum_{n=0}^{N-1}{\left\{\left[y\left(m+i,n+j\right)-\bar{y}\right]\right\}}^{2}}}$$ (5)

$\overset{-}{x}$ was the mean pixel value of rectangular box *x*(*m*, *n*), while $\overset{-}{y}$ was the mean pixel value of rectangular box *y*(*m*, *n*).

Gray-level co-occurrence matrix was applied to extract muscle texture features in USBI images. The extracted features were angular second moment (ASM), contrast, and homogeneity (HOM), which were computed as Eqs (6)–(8) below.


$$ASM=\sum \sum {\left[U\left(i,j\right)\right]}^{2}$$ (6)

$$Contrast=\sum \sum {\left(i-j\right)}^{2}U\left(i,j\right)$$ (7)

$$HOM=\sum_{i}\sum_{j}\frac{1}{1+{\left(i-j\right)}^{2}}U\left(i,j\right)$$ (8)

In the above equations, *U*(*i*, *j*) represented the gray-level co-occurrence matrix. ASM could reflect the uniformity of image gray distribution and the thickness of texture features. Contrast reflected the sharpness of the image and the depth of the texture features. HOM could reflect the homogeneity and local uniformity of texture features in an image.

### 3.5 Observed indicators

In a relaxed condition, the lateral femoral skin was wiped with alcohol and the electrodes were fixed to the lateral femoral muscle at least 2 cm apart. For SEMG signal acquisition, the sampling frequency was 1,000 Hz, the high common-mode rejection ratio was 110 dB, the noise level was less than 3.5 μV, and the band-pass filtering was 20–500 Hz. Subsequently, denoising and feature extraction of SEMG signals were carried out, and a model was developed to identify muscle fatigue state. According to the processed SEMG signals, the time-domain indicator were integrated electromyography (IEMG) and root mean square (RMS) amplitude, while the frequency domain indicators consisted of the mean power frequency (MPF).

Ultrasound scanner was applied for ultrasonic image scans of muscles, focusing on the morphological changes of surface muscles and deep muscles. The thicknesses of surface muscles and deep muscles as well as the total thickness were calculated, respectively. In USBI images acquired, speckle noise should be suppressed and high-frequency noise should be removed. The gray-level co-incidence matrix was adopted to extract muscle texture features in ultrasound.

3 mL of the subjects’ venous blood was collected before and after exercise, respectively. After centrifugation, routine indicators such as hemoglobin concentration (HB), red blood cell count (RBC), and mean corpuscular volume of red blood cells (MCV) were detected. The concentration of BLA was determined by Barker-Summerson method. Blood malondialdehyde (MDA) concentration was detected by enzyme linked immunosorbent assay kit. Blood creatine phosphokinase (CPK) concentration was also detected using the creatine kinase coupling with ultraviolet dual kit.

## 4. Evaluation results

### 4.1 Performance evaluation of SEMG signal denoising

**Fig 2:**
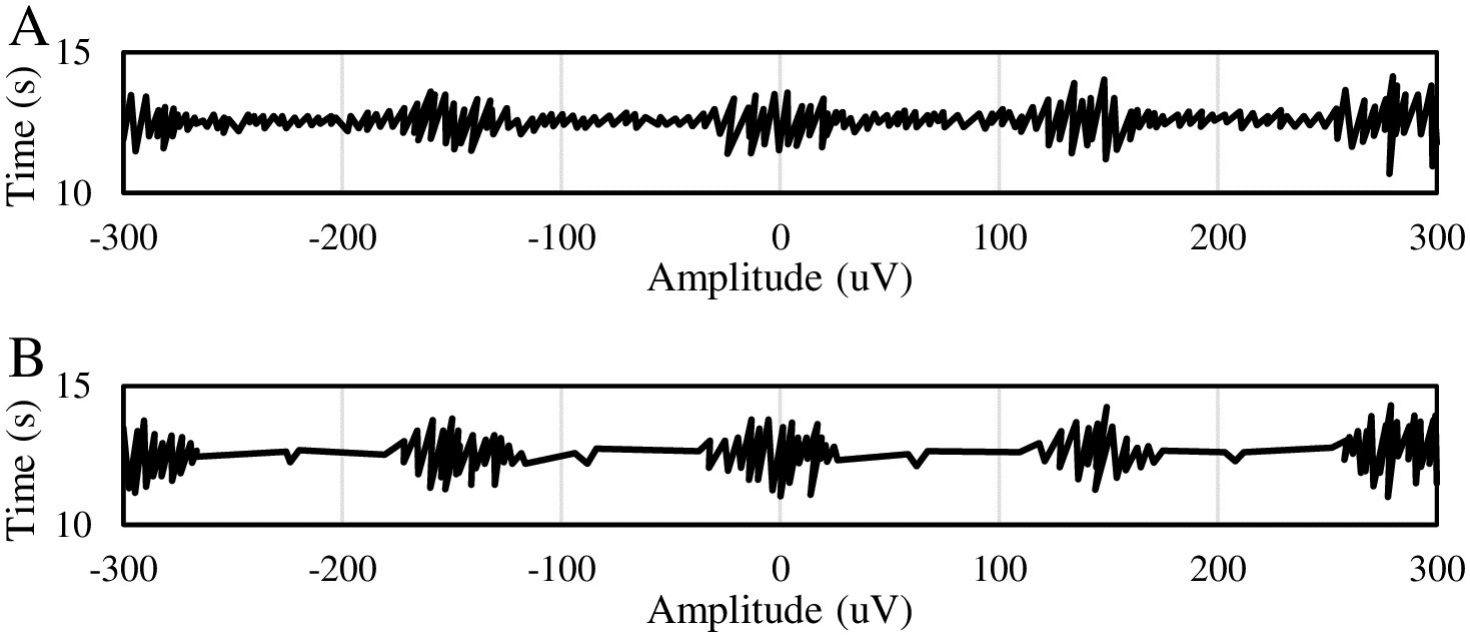
Subjective evaluation of SEMG signal before and after denoising. (A) SEMG signal with noise; (B) SEMG signal after denoising.

The denoising effect on SEMG signals by wavelet threshold function was analyzed as shown in Fig 2. Fig 2A was the original SEMG signal, while Fig 2B was the SEMG signal after denoising by wavelet threshold function. Subjectively, after denoised by wavelet threshold function, SEMG signal had a high coincidence degree with the original signal, but the noises in the signal were obviously removed.

Signal-to-noise ratio (SNR) and RMS error (RMSE) were taken to evaluate the denoising efficiency on SEMG signals by wavelet threshold function, as displayed in Table 1. The denoising performance of the wavelet threshold function with hard threshold, soft threshold, and improved threshold was compared. SNR of different threshold functions were 37.145 dB, 38.029 dB, and 39.725 dB, respectively; RMSE were 5.673, 4.984, and 3.721, respectively. For the wavelet threshold function with the improved threshold used in this work in denoising SEMG signal, the SNR was the greatest and the RMSE was the smallest.

**Table 1:** Objective evaluation of surface EMG signal before and after denoising.

	SNR	RMSE
Hard threshold	37.145	5.673
Soft threshold	38.029	4.984
Improve threshold	39.725	3.721

### 4.2 Performance evaluation of muscle fatigue recognition model

SEMG data of 20 subjects were collected before and after the experiment, and 1,000 pieces of data were collected for each. Then the signal data were randomly divided into test set and verification set, including 600 and 1,400 pieces, respectively in a ratio of 7:3. Firstly, the test set was utilized to train the classification and recognition models support vector machine (SVM), artificial back propagation neural network (BPNN), convolutional neural network (CNN), and LSTM. After training, the verification set was utilized to compare the accuracy, sensitivity, specificity, precision, and recognition time of each model for muscle fatigue recognition, as in Table 2. The accuracy, sensitivity, specificity, precision, and recognition time of SVM model were 86.25%, 86.47%, 86.04%, 85.69%, and 26.83 minutes, respectively. Those indicators of BPNN model were 88.70%, 89.49%, 87.91%, 88.08%, and 19.07 minutes, respectively. Those of CNN model were 89.55%, 90.86%, 88.25%, 88.47%, and 17.24 minutes, respectively; and those of LSTM model were 94.10%, 94.43%, 93.75%, 94.07%, and 23.12 min, respectively. The accuracy, sensitivity, specificity, and precision of the LSTM model for muscle fatigue recognition were higher than those of SVM, BPNN, and CNN models, but the recognition time was relatively longer.

**Table 2:** Objective evaluation of muscle fatigue recognition model.

	SVM	BPNN	CNN	LSTM
Accuracy (%)	86.25	88.7	89.55	94.1
Sensitivity (%)	86.47	89.49	90.86	94.43
Specificity (%)	86.04	87.91	88.25	93.75
Precision (%)	85.69	88.08	88.47	94.07
Time (min)	26.83	19.07	17.24	23.12

### 4.3 Performance of serological indicators of VEDs in relieving EIF of muscles

**Fig 3:**
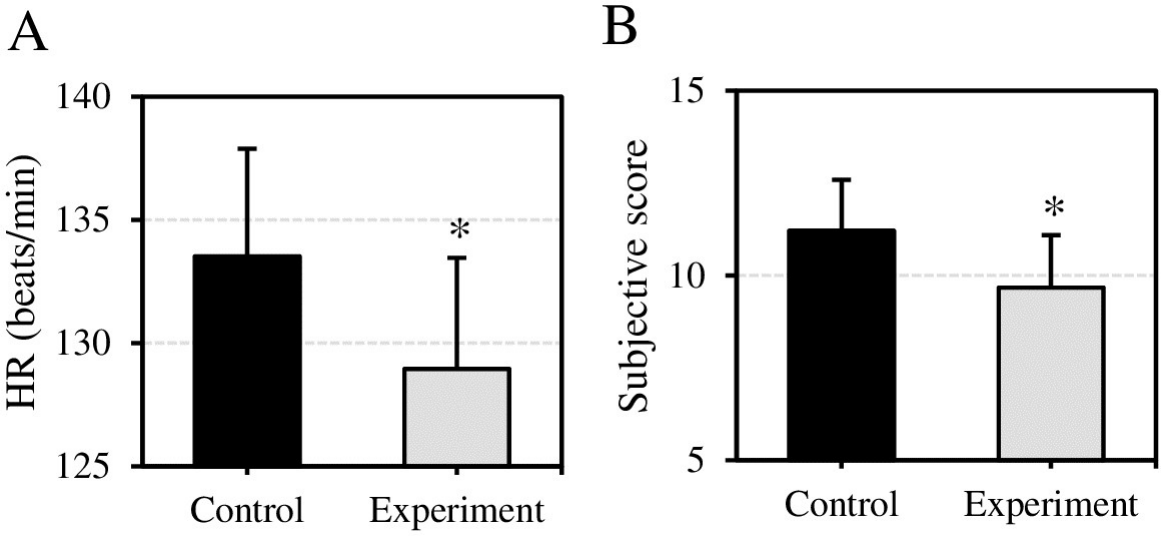
Comparison of subjects’ heart rate and subjective score after exercise. (A) Comparison of heart rate after exercise; (B) Comparison of subjective scores after exercise. Compared with those in Ctrl group, *P<0.05.

The effect of VEDs before exercise on the physiological indicators of these subjects after exercise was evaluated in Fig 3. The heart rate of the subjects in Ctrl group and Expt group after exercise was (133.52±4.37) beats/min and (128.95±4.51) beats/min, respectively. The subjective exercise scores were 11.21±1.38 and 9.67±1.42, respectively. Compared with Ctrl group, the heart rate and subjective exercise score of subjects in Expt group were highly decreased, showing differences being statistically significant (*P*<0.05).

**Fig 4:**
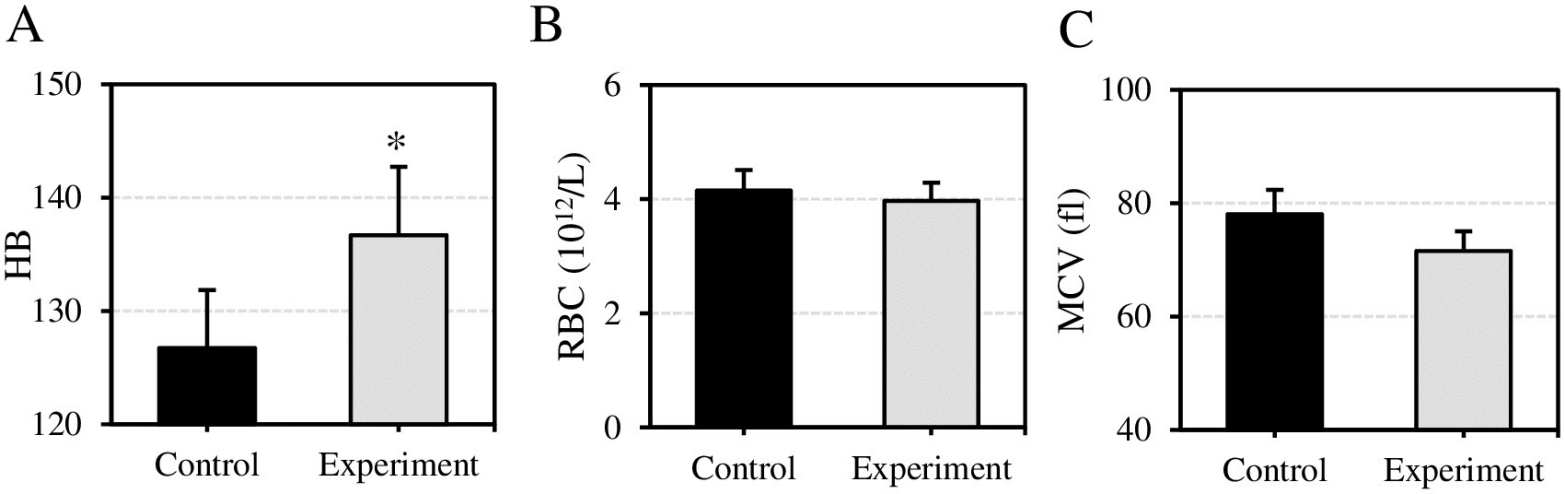
Comparison of HB, RBC, and MCV of subjects after exercise. (A) Comparison of HB of subjects; (B) Comparison of RBC in subjects’ blood; (C) Comparison of subjects’ MVC. Compared with Ctrl group, *P<0.05.

The effect of VEDs before exercise on routine serological indicators of subjects after exercise were assessed as in Fig 4. HB levels in Ctrl group and Expt group were (126.73±5.11) and (136.69±6.03), respectively. RBC was (4.15±0.36)×10^12^/L and (3.97±0.32)×10^12^/L, respectively. MCV reached (78.05±4.32) fl and (71.56±3.48) fl, respectively. Compared to Ctrl group, the HB of Expt group was greatly increased with a statistically significant difference (*P*<0.05). There was not any significant difference in RBC and MCV between Ctrl and Expt groups (*P*>0.05).

**Fig 5:**
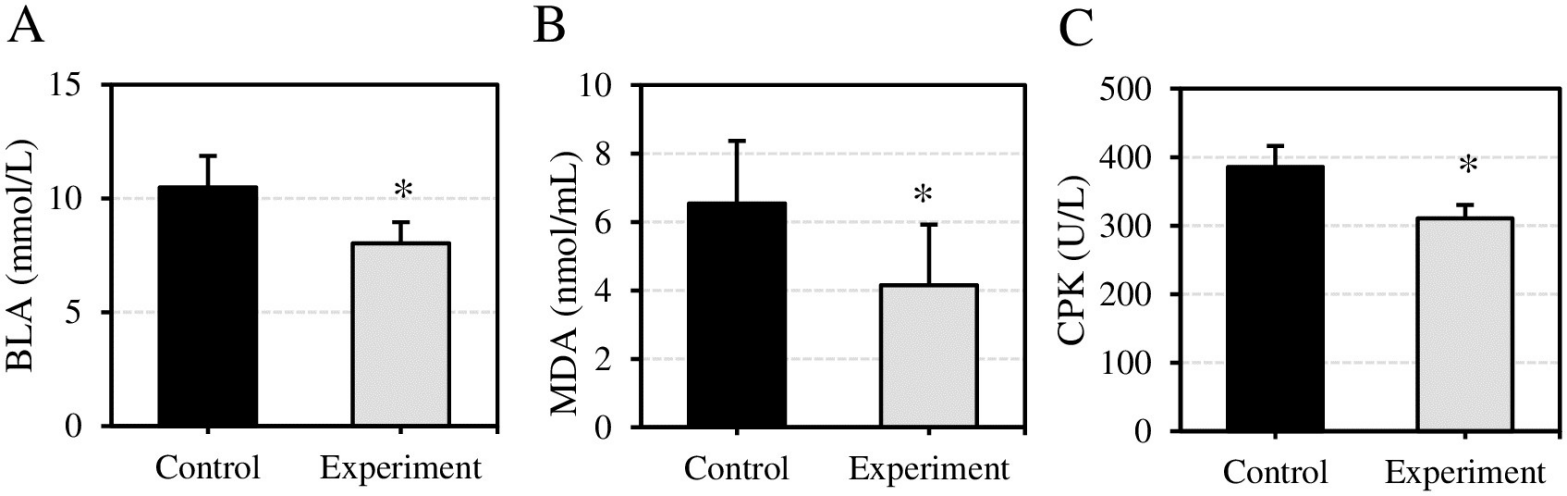
Comparison of BLA, MDA, and CPK levels after exercise. (A) Comparison of BLA levels of subjects; (B) Comparison of MDA levels in subjects’ blood; (C) Comparison of CPK levels in subjects’ blood. Compared with Ctrl group, *P<0.05.

The effect of VEDs before exercise on the related indicators of serological stress after exercise was assessed in Fig 5. The BLA levels after exercise were (10.49±1.38) mmol/L and (8.03±0.93) mmol/L in Ctrl group and Expt group, respectively. MDA levels were (6.54±1.83) nmol/mL and (4.15±1.77) nmol/mL, respectively; and CPK levels were (385.67±30.72) U/L and (310.52±19.61) U/L, respectively. Compared with Ctrl group, the levels of BLA, MDA, and CPK in Expt group were considerably decreased with statistical significance (*P*<0.05).

### 4.4 Electromyographic parameter performance of VEDs in relieving EIF of muscle

**Fig 6:**
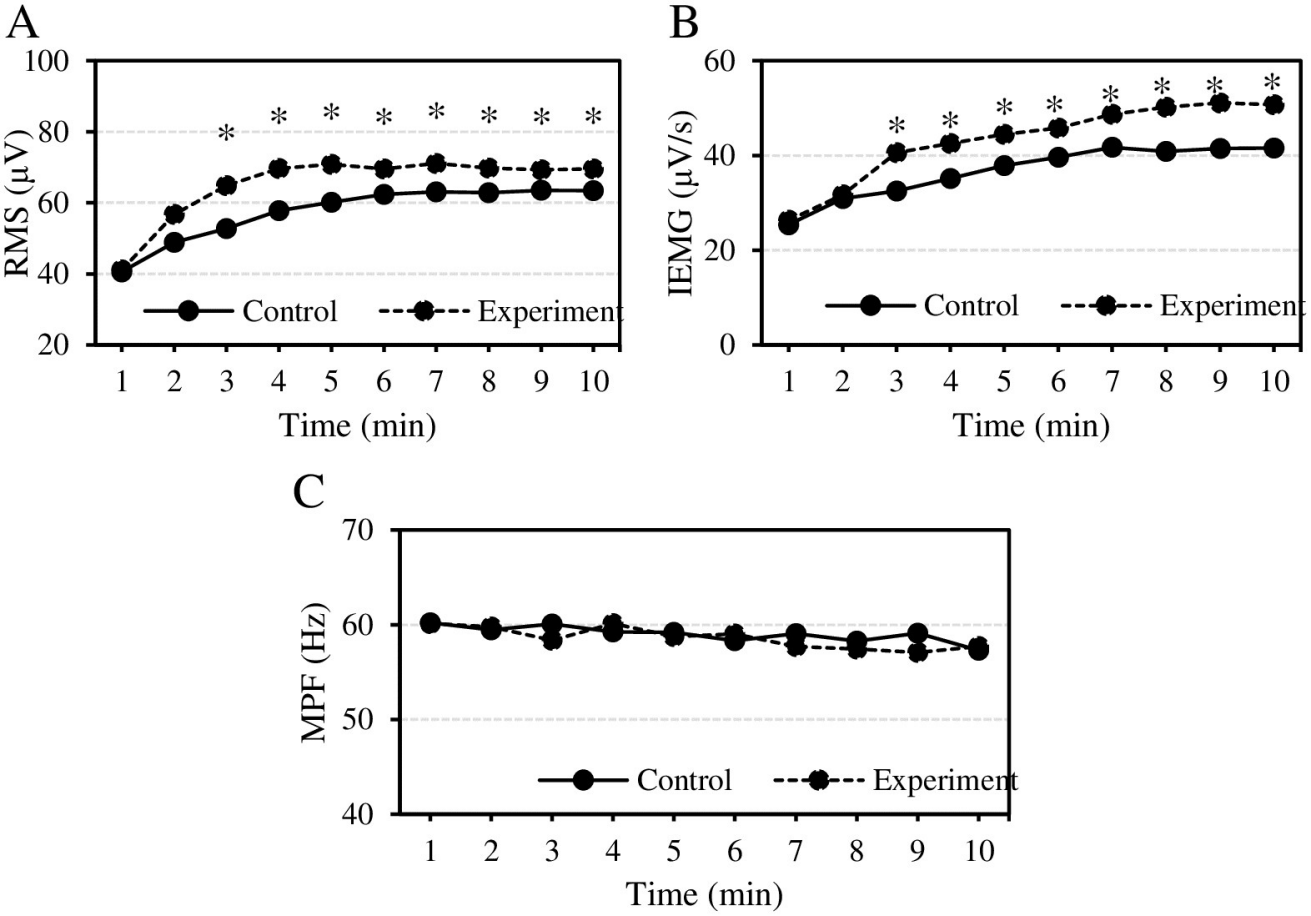
Comparison of electromyographic parameters at each time point after exercise. (A) Changes in the time domain feature RMS; (B) IEMG changes in time domain features of subjects’ SEMG signals; (C) Changes of MPF in the frequency domain. Compared to Ctrl group, *P<0.05.

The changes of electromyographic parameters of subjects at different time points after exercise were evaluated as displayed in Fig 6. With the increase of exercise time, the time domain features of SEMG signals (RMS and IEMG) in both Ctrl group and Expt group gradually increased, while the frequency domain feature of MPF changed little. 3, 4, 5, 6, 7, 8, 9, and 10 minutes after exercise, the RMS and IEMG levels in Expt group were always remarkably higher than those in Ctrl group; the difference was also statistically significant all the time (*P*<0.05). None of significant differences were shown in MPF level between groups (*P*>0.05).

### 4.5 USBI image parameter performance of VEDs in relieving EIF of muscle

Response signal amplitude values of muscle USBI images were collected at different time points after exercise, which were compared in Table 3. With the increase of exercise time, the mean value of response signal amplitude of muscle USBI images in both Ctrl and Expt groups decreased gradually. Compared to Ctrl group, the mean amplitudes of muscle USBI image response signal in Expt group decreased observably 30 s, 60 s, 120 s, 240 s, and 480 s after exercise, with differences having statistical significance (*P*<0.05).

**Table 3:** Comparison of mean amplitudes of ultrasonic biological image response signals at different time points after subject exercise.

Range	Control group (n = 10 cases)	Experimental group (n = 10 cases)
5	0.0092	0.0097
15	0.0071	0.0085
30	0.0065	0.008*
60	0.005	0.0074*
120	0.0043	0.0067*
240	0.0023	0.0053*
480	0.001	0.004*

Note:

* meant the difference was great with *P* < 0.05 in contrast to the value in the control group.

**Fig 7:**
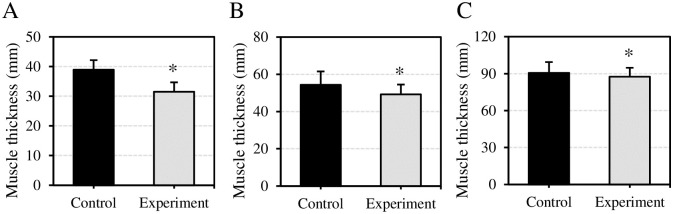
Comparison of USBI image parameters of muscles after exercise. (A) Comparison of surface muscle thickness of subjects; (B) Comparison of deep muscle thickness of subjects; (C) Comparison of total muscle thickness of subjects. Compared with Ctrl group, *P<0.05.

The effect of VEDs before exercise on USBI image parameters of muscles after exercise was evaluated in Fig 7. The surface muscle thickness of subjects after exercise in Ctrl group and Expt group was (38.92±3.23) mm and (31.51±3.16) mm, respectively. The deep muscle thickness was (54.35±7.17) mm and (49.20±5.28) mm, respectively. The total muscle thickness reached (90.61±8.79) mm and (87.52±7.24) mm, respectively. Compared to Ctrl group, the USBI image parameters of surface, deep, and total muscle thickness in Expt group after exercise were notably reduced, showing differences of statistical significance (*P*<0.05).

The effect of VEDs before exercise on the texture features of USBI images of muscles of subjects after exercise was evaluated in Table 4. ASM after exercise was (0.54±0.08) in Ctrl group and (0.62±0.10) in Expt group. Contrast was (-0.51±0.06) and (-0.33±0.12), respectively; HOM was 0.62±0.13 and 0.41±0.09, respectively. Compared to Ctrl group, the texture features ASM and contrast of muscle USBI images in Expt group increased notably, while HOM decreased observably, showing differences having statistical significance (*P*<0.05).

**Table 4:** Comparison of texture features of USBI images of muscles after exercise.

	ASM	CON	HOM
Control group (n = 10 cases)	0.54 ± 0.08	-0.51 ± 0.06	0.62 ± 0.13
Experimental group (n = 10 cases)	0.62 ± 0.1*	-0.33 ± 0.12*	0.41 ± 0.09*

Note:

* meant the difference was great with *P* < 0.05 in contrast to the value in the control group.

## 5. Discussion

The consumption of glycogens in body is the major factor leading to EIF. With the increase of exercise time and intensity, the body will consume a great number of glycogens, thus promoting the excessive generation and accumulation of BLA, which will cause EIF and muscle soreness [24]. Appropriate supplement of energy drinks can effectively maintain the stability of the body’s HB and blood glucose level, promote the improvement of skeletal muscle function, and ultimately avoid central fatigue [25]. This work found that the heart rate of the subjects supplemented with VED was considerably lower than that of the purified water drinkers after exercise, and the serum HB level increased. This indicated that the supplementation of VEDs could ensure the stability of HB of subjects after exercise, prevent its great reduction, and improve the physical reserve of the subjects. The body generates BLA through glycolysis under hypoxia, which can be used to reflect the adaptability of the body to salt metabolism [26]. MDA is the material basis of cell membrane damage; it can indirectly reflect the damage degree of oxygen free radicals to cells and can also be taken to evaluate the severity of EIF [27]. CPK is an important enzyme that regulates cellular energy reserve and is mainly distributed in skeletal muscle [28]. Cheng et al. (2019) [29] showed that long-term and high-intensity load exercise would lead to an increase in the concentration of CPK in human blood. In this work, the levels of BLA, CPK, and MDA after exercise of the subjects supplemented with VEDs were much lower than those of purified water drinkers. The results suggested that VEDs could reduce the production rate of BLA and the content of CPK in serum, lower the fatigue caused by the accumulation of BLA, and thus delay EIF. MDA can reflect the oxidative damage caused by aerobic quantitative load exercise [30]. VEDs can reduce the level of serum MDA after exercise, indicating that the drink could reduce the severity of lipid peroxidation and oxidative damage.

Accurate identification of muscle fatigue state is of great significance to kinematics and rehabilitation medicine. In addition to serological indicators, SEMG signal and USBI technology are also widely used in the assessment of muscle status, muscle strength, and endurance [31]. EIF manifests a reversible decrease in the maximum voluntary contraction ability and maximum output function of muscles [32]. In this work, the wavelet threshold function was applied to denoise the SEMG signals, and then the LSTM model was utilized to identify muscle fatigue state. The recognition efficiency of this model was superior to SVM [33], BPNN [34], and CNN [35] models. The LSTM model included memory units, which was allowed to effectively capture key information and retain it for a certain period. Therefore, the network could remember the changes of SEMG signal features in the process of muscle fatigue, thus improving the recognition performance [36]. RMS and IEMG are characteristic parameters of SEMG signals, which can reflect the changes of electromyographic amplitude. There was also a synchronization of RMS with the excitability of the dominant motor unit of cerebral cortex [37]. IEMG reflects the number of motor units recruited with the change of muscle strength [38]. IEMG can increase with the deepening of muscle contraction fatigue, indicating that not all muscle fibers in the same muscle are involved in contraction. This can reflect the total number of muscle fiber units participating in exercise [39]. The results of this work demonstrated that RMS and IEMG of the subjects after drinking VEDs gradually increased with prolonging exercise time, higher than those of purified water drinking. Meanwhile, the inflection point of IEMG was before that of the purified water drinking group. It was illustrated that VEDs could better mobilize the movement of muscle fibers in the unit muscle, which was very important for improving the severity of muscle fatigue.

USBI images are used for the detection of muscle tissue and can also be used to calculate muscle parameters such as muscle bundle length and muscle thickness [40]. In this work, USBI images were utilized to evaluate the effect of VEDs and purified water on muscle EIF. The surface muscle thickness, deep muscle thickness, and total muscle thickness of VED drinking subjects after exercise were smaller than those of purified water drinking group. The greater the muscle thickness, the more intense the muscle contraction, often requiring more energy consumption [41]. The results suggested that the VED could reduce the muscle contraction intensity of the subjects, and then reduce the energy consumption, which was beneficial to reduce the severity of EIF. Image texture information can be taken to diagnose tissue lesions. To segment an image effectively, the spatial frequency domain and the mean gray value are often used for evaluation of image texture features. Gray-level co-occurrence matrix combines spatial location distribution and brightness distribution characteristics. Compared with other feature extraction algorithms, gray-level co-occurrence matrix can extract more brightness information [42]. In this work, gray-level co-occurrence matrix was adopted to extract ASM, contrast, and HOM features in muscle USBI images. The ASM and contrast texture features of muscle USBI images after exercise of VED drinkers were observably higher than those of purified water drinkers, while the HOM was considerably lower than that of purified water drinkers. When muscle contraction is intense, image texture feature parameters can reflect the severity of muscle fatigue [43]. ASM reflects the uniformity of gray distribution and texture thickness in an image [44]. Contrast can reflect the clarity of the image, and the larger the value, the clearer the visual effect will be [45]. HOM reflects the homogeneity of image texture, and the larger the value, the more uniform the local texture of the image [46]. VEDs could affect the exercise state of muscles, and then affected the ratio of muscle texture features; it was very important for improving the muscle state of EIF.

## 6. Conclusion

In summary, SEMG signals and USBI technology could be applied to monitor and evaluate muscle fatigue. VEDs could reduce the concentration of a variety of fatigue-related indicators in the blood, inhibit excessive muscle contraction. Thus, it could improve the state of EIF to a certain extent and promote the recovery of EIF. This work evaluated the effects of VEDs on the EIF state merely without further exploration of the influencing mechanism. It would be expected to prepare animal models of acute exhaustive exercise, and to analyze the effect of VEDs on exercise ability. In conclusion, the results offered a reference for understanding the effect of VEDs on resisting EIF and further exploring the biological features of EIF muscles.

## Supporting information

S1 Dataset (XLSX)
